# The Opportunity for High-Performance Biomaterials from Methane

**DOI:** 10.3390/microorganisms4010011

**Published:** 2016-02-03

**Authors:** Peter James Strong, Bronwyn Laycock, Syarifah Nuraqmar Syed Mahamud, Paul Douglas Jensen, Paul Andrew Lant, Gene Tyson, Steven Pratt

**Affiliations:** 1 Centre for Solid Waste Bioprocessing, School of Civil Engineering and School of Chemical Engineering, The University of Queensland, Brisbane, Queensland 4072, Australia; 2 School of Chemical Engineering, The University of Queensland, St. Lucia, Brisbane, Queensland 4072, Australia; b.laycock@uq.edu.au (B.L.); aqmar.syedmahamud@uq.edu.au (S.N.S.M.); paul.lant@uq.edu.au (P.A.L.); s.pratt@uq.edu.au (S.P.); 3 Advanced Water Management Centre, The University of Queensland, Brisbane, Queensland 4072, Australia; p.jensen@awmc.uq.edu.au; 4 Australian Centre for Ecogenomics, The University of Queensland, Brisbane, Queensland 4072, Australia; g.tyson@uq.edu.au

**Keywords:** PHA, PHB, PHBV, methane, syngas methanotroph, gas fermentation, biopolymer

## Abstract

Polyhydroxyalkanoate (PHA) biopolymers are widely recognised as outstanding candidates to replace conventional petroleum-derived polymers. Their mechanical properties are good and can be tailored through copolymer composition, they are biodegradable, and unlike many alternatives, they do not rely on oil-based feedstocks. Further, they are the only commodity polymer that can be synthesised intracellularly, ensuring stereoregularity and high molecular weight. However, despite offering enormous potential for many years, they are still not making a significant impact. This is broadly because commercial uptake has been limited by variable performance (inconsistent polymer properties) and high production costs of the raw polymer. Additionally, the main type of PHA produced naturally is poly-3-hydroxybutyrate (PHB), which has limited scope due to its brittle nature and low thermal stability, as well as its tendency to embrittle over time. Production cost is strongly impacted by the type of the feedstock used. In this article we consider: the production of PHAs from methanotrophs using methane as a cost-effective substrate; the use of mixed cultures, as opposed to pure strains; and strategies to generate a poly(3-hydroxybutyrate-*co*-3-hydroxyvalerate) copolymer (PHBV), which has more desirable qualities such as toughness and elasticity.

## 1. Introduction

Polyhydroxyalkanoate (PHA) biopolymers are high molecular weight, stereoregular, linear thermoplastic polymers that are naturally produced by bacteria in an aqueous environment. Their mechanical properties are good, they are biodegradable, and, unlike many alternatives, they do not rely on oil-based feedstocks [1,2]. PHAs are intra-cellular storage granules that serve as a source of carbon (C), energy, or reducing-power and may comprise up to 90% of a microbe’s dry weight [3,4]. The industrial production of poly-3-hydroxybutyrate (PHB), the simplest and most widely produced form of PHA, involves accumulation of polymer in pure strains using plant-derived carbon sources, typically sugars, as the feedstock. PHA accumulation is generally enhanced by culturing the microbes under a nutrient limitation in the presence of excess carbon. The availability of alternative carbon sources, pH, temperature, oxygen, methane, carbon dioxide, macronutrients (nitrogen, phosphorus, sulphur, potassium, magnesium sodium), and trace metals (copper, iron, zinc, manganese, and/or cobalt) can all affect PHA yield and quality [5,6,7].

However, the production of PHA by conventional means also uses expensive refined substrates and requires sterilisation, limiting widespread commercialisation [8]. Techno-economic studies have shown that a major cost of pure culture production is the carbon feedstock, estimated to be up to 40% of the product cost [9,10,11]. Consequently, many research groups are investigating the potential of using waste streams for PHA production, such as dairy whey waste, waste lipids, sugar industry waste streams, agricultural crop residues, petrochemical waste, syngas and glycerol [12,13]. The problems with waste streams, however, are their limited abundance and distributed nature. In contrast, methane is a cheap, abundant and widely available carbon source. Additionally, the robust, self-regulating nature of mixed methanotrophic cultures [14] offers the opportunity to operate under non-sterile conditions, thereby reducing operating costs on an industrial scale. Over 300 bacterial strains, including methanotrophs, have shown potential to synthesise and store PHB [15].

Methane is the principal component of natural gas, is abundantly available in many oilfields and coal deposits, and is widely produced during the biological degradation of organic matter, either in engineered processes or natural environments. Methane is considered to be the second most abundant greenhouse gas (GHG) after carbon dioxide, contributing to 18% of the total atmospheric radiative forcing [16,17]. Globally, over 60% of total methane emissions are anthropogenic emissions, the majority of which are the result of microbial metabolism [16,18]. Microbial methane production occurs during the anaerobic biodegradation of complex organic matter. Complex molecules and solids are solubilised (hydrolysis), degraded to various organic acids, such as acetic, propionic, butyric acids (acidogenesis), converted to acetic acid (acetogenesis), and finally transformed into methane during methanogenesis [19].

Microbial methane production and capture is an established and sustainable strategy for the treatment of organic waste streams in a number of industries (e.g., municipal solid waste, wastewater treatment, brewing, food processing, agriculture, crop residues). When captured, microbially-produced methane is a potentially valuable and renewable resource [20]. Significant sources of renewable methane that are either captured, or present the opportunity for capture and reuse, include landfills (38 Mt/year), wastewater treatment (21 Mt/year), agriculture (11–30 Mt/year), and biomass (10 Mt/year). However, with natural gas alone, the World Bank estimates wastage of 92 Mt/year that is flared or vented [21] which represents an enormous opportunity for converting methane to higher value products [18].

The rapid expansion of global methane production and capture, both in the form of natural gas and generation of methane-rich biogas, has improved the accessibility of methane on the global market and consequently reduced the commodity price. This has generated increased interest in methane as a carbon source for novel, value-added processes. The thermochemical processing of methane can produce syngas and methanol [22,23], which are robust processes, but require large capital investments that can exceed a billion USD and prohibit many applications. There has been growing interest in the biological conversion of C_1_ compounds to fine and commodity chemicals due to recent economic changes and the mind shift towards sustainably-produced chemicals. 

**Figure 1:**
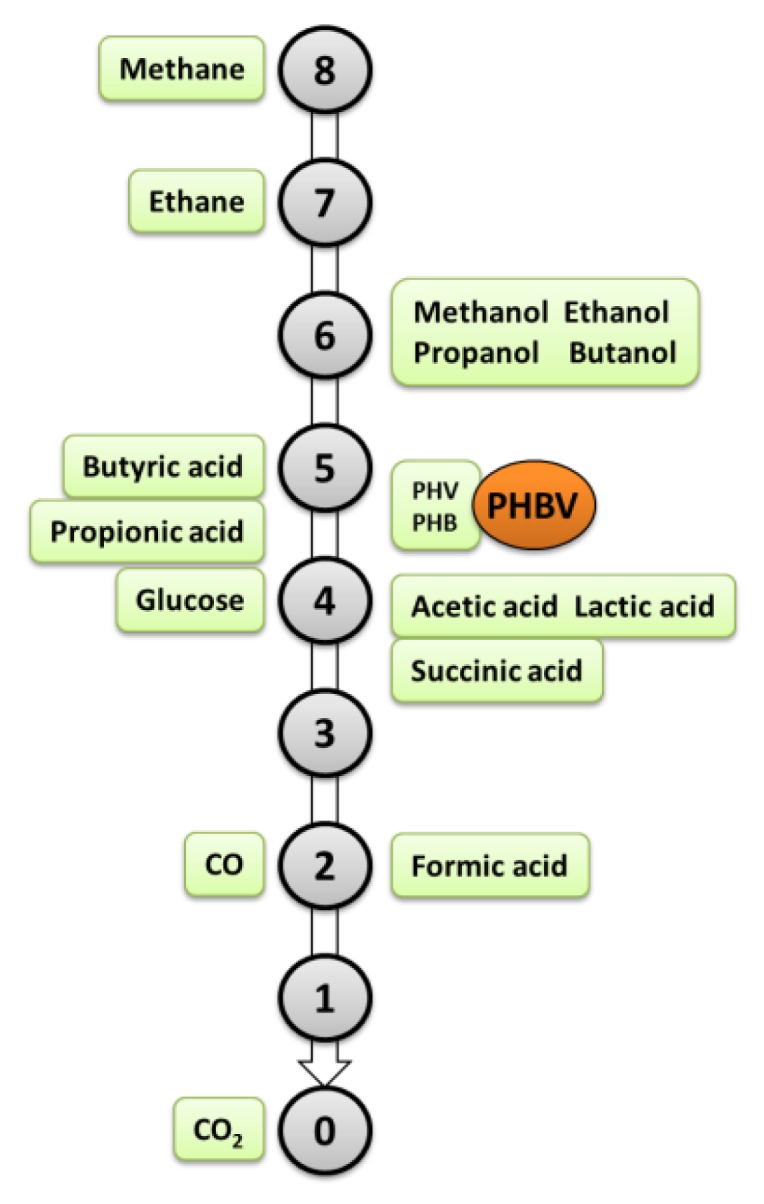
Degree of reduction of methane relative to other feedstocks, metabolites, PHB (CH_1.5_O_0.5_: 4.5), and PHV (CH_1.6_O_0.4_: 4.8). The degree of reduction is of a measure of the oxidation potential, with high oxidation potential indicating higher potential for energy release upon oxidation. The figure is modified from Kracke and Kromer [29] and Pratt [30].

Pure methane is an energy-rich feedstock with an energy density of 55.7 MJ/kg at 1.013 bar, 15 °C [24]. Figure 1 illustrates the degree of reduction of various organic feedstocks, metabolites, and products. It shows that the oxidation potential of methane (the energy content) is greater than that of the product PHB, greater than the widely-used alternative feedstocks, e.g., sugar (glucose) and organic acids (acetic, propionic and butyric acids), and much greater than CO_2_. Using CO_2_ as a feedstock to generate PHB would, thus, be akin to “pushing up hill”, requiring significant electron donor supplementation. This translates to more favourable energy balances when using methane. For example, recent life cycle assessments of biogas generated at wastewater treatment plants and landfills and used as a feedstock for PHA production indicate a lower energy requirement for PHB production from methane (37 MJ/kg PHB from biogas) compared to PHB production from alternative renewable substrates (42 MJ/kg PHB from corn-derived sugar) [25]. There is also significant potential for enhanced carbon sequestration (at ~2 kg CO_2_ equivalents fixed/kg methane compared to 0.1 kg CO_2_ equivalents fixed/kg of corn-derived sugar). What is more, the cost of methane is around $520/tonne carbon, based on a commercial price for natural gas in the United States of US $7.74/thousand cubic feet [26]. If the fraction of carbon converted to PHA is considered, at, for example, a PHB yield of around 0.55 g/g methane as reported by Wendlandt *et al.* [27], methane has potentially strong economic benefits [28]. 

**Figure 2:**
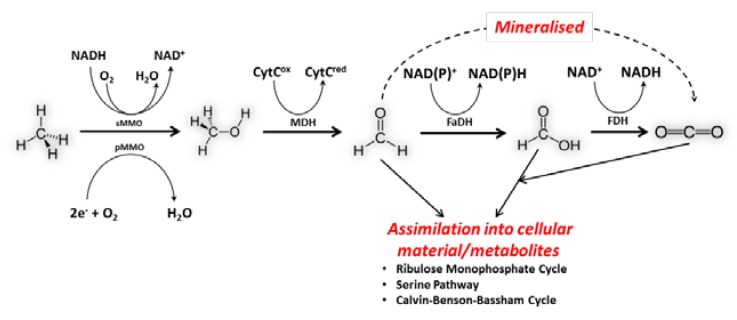
Generalised reaction scheme for methane oxidation via methanotrophs. Adapted from Kalyuzhnaya *et al.* [32] and Hanson and Hanson [31]; sMMO: soluble methane mono-oxygenase, pMMO: particulate methane mono-oxygenase, MDH: methanol dehydrogenase, FaDH: formaldehyde dehydrogenase, FDH: formate dehydrogenase and CytC: cytochrome C.

The methanotrophic conversion of methane into biomass and CO_2_ plays a large part in regulating the global methane cycle by serving as a biological CH_4_ sink [31]. Methane monooxygenase is the key enzyme responsible for CH_4_ oxidation to methanol, which is further oxidised to CO_2_ to regenerate reducing equivalents, or assimilated into cellular components (Figure 2). Traditionally, these bacteria were classified as Type I (γ-proteobacteria) or Type II (α-proteobacteria) methanotrophs, primarily according to their use of the ribulose monophosphate pathway (Type I) or serine (Type II) pathways for formaldehyde assimilation). They were further subdivided into a Type X group, which contained biochemical capabilities of Type I and II. Methanotrophs are now typically grouped as γ-proteobacteria or α-proteobacteria (where Type X is a subdivision of γ-proteobacteria). A recently-discovered thermophilic subset, Verrucomicrobia (*Methylacidiphilum* and *Methylomirabilis* spp.), was added to the group (see Kalyuzhnaya *et al.* [32] and Strong *et al.* [33] and references therein).

Certain methanotrophs can synthesise the PHB homopolymer from methane under nutrient limited conditions [6]. The general ability of methanotrophic and methylotrophic bacteria to produce PHB was described for several strains as early as 1970, in the seminal work by Whittenbury, *et al.* [34], and has been of consistent interest for decades [6,35,36,37,38,39]. The use of methanotrophic bacteria is viewed as a powerful route to the microbial production of PHB from methane, as it provides a collective solution for three major environmental problems:

(1) A potential approach for carbon sequestration and GHGs emission reduction;
(2) Production of biodegradable polymers for replacing conventional fossil fuel-derived plastics;
(3) Reducing the use of organic carbon sources such as sugars for PHB production [6].

Overall, therefore, this review seeks to provide an overview of the current state of research into the production of PHAs from methanotrophs using methane as a cost-effective substrate as well as the use of mixed cultures as opposed to pure strains and strategies to generate a poly(3-hydroxybutyrate-*co*-3-hydroxyvalerate) copolymer (PHBV), which has more desirable qualities, such as toughness and elasticity.

## 2. Metabolism of Methanotrophs and Biosynthesis of PHB from Methane

It is important to consider the biochemical capabilities of methanotrophs and their microbial consortia with regard to biopolymer synthesis. This enables better selection of culture conditions that favour PHB-producing strains, or can establish whether the mixed culture is capable of PHB synthesis at all. The first step in aerobic methane catalysis consumes methane, oxygen, and reducing equivalents. Methane mono-oxygenase breaks the O-O bond in the oxygen molecule by using two reducing equivalents [31]. One of the oxygen atoms is incorporated into methane to form methanol, while the second is converted to water. Methane mono-oxygenase can occur as a particulate, membrane-bound form (pMMO) or in a soluble form (sMMO). The sMMO requires NAD(P)H and O_2_ to convert methane to methanol in the following manner: CH_4_ + NAD(P)H + H^+^ + O_2_ = CH_3_OH + NAD(P)^+^ + H_2_O, while the pMMO requires cytochromes b559/569 or c553 or artificial reductants such as duroquinol and NADH to complete the reaction [40]. However, despite a great deal of effort in this area, the physiological source of the electron donor to the pMMO remains unresolved [32]. The pMMO and the ammonia mono-oxygenase share many similarities and an endogenenous quinol may serve as a reductant for both enzymes, and may potentially abstract electrons from the subsequent methanol oxidation via the periplasmic PQQ-linked methanol dehydrogenase, which is coupled to a cytochrome c [41]. Direct coupling of pMMO and methanol dehydrogenase catalysis is supported by their close proximity and reports of a super-complex of the two enzymes [42,43,44,45]. The ability to convert methane into organic acids and H_2_ under oxygen-limiting conditions suggests that pMMO can use electron donors other than NADH [46]. Considering the range of metabolic capabilities of the methane-oxidising bacteria, it is likely that there are multiple systems for electron abstraction, and that these may vary between Gammaproteobacteria, Alphaproteobacteria, and Verrucomicrobia. 

**Figure 3:**
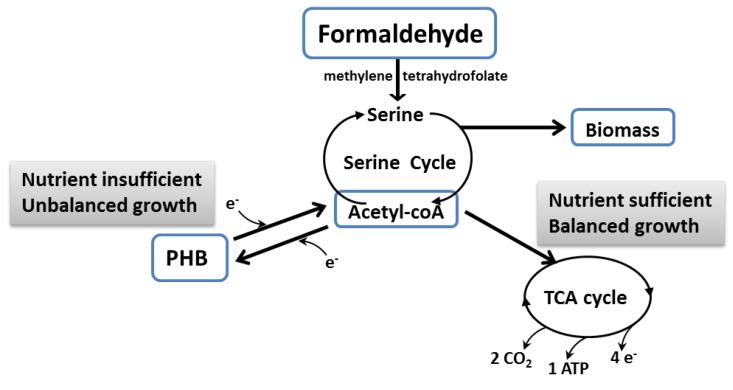
Schematic indicating carbon flow from the serine cycle with regard to balanced or unbalanced growth (adapted by combining Karthikeyan *et al.* [6] and Pieja *et al.* [54]).

After methane oxidation, methanol is oxidised to formaldehyde (by methanol dehydrogenase), which is the central metabolite in the anabolic and catabolic pathways (Figure 2). In the catabolic pathway, formaldehyde is further converted to formate and then to CO_2_, regenerating two NADH. Formaldehyde is, in turn, assimilated using different pathways in α-proteobacteria or γ-proteobacteria. Typically, γ-proteobacteria use the ribulose monophosphate (RuMP) pathway, in which the 3-hexulose-phosphate-synthase (HPS) catalyses formaldehyde conversion into (*D*-arabino)-3-hexulose-6-phosphate. *D*-arabino-3-hexulose-6-phosphate is then converted into different intermediates and assimilated as cell biomass or oxidised to CO_2_ via an incomplete TCA cycle. The α-proteobacteria typically assimilate formaldehyde using the serine pathway [47]. Formaldehyde oxidation is activated by the pterin co-factor catalysed by methylene tetrahydrofolate, an enzyme that combines formaldehyde with glycine to generate serine [48,49]. Serine then either enters the TCA cycle under nutrient-sufficient balanced growth, or the PHB cycle under nutrient-deficient conditions (Figure 3). 

PHB formation in methanotrophs starts from acetyl-CoA molecules and proceeds via the enzyme-mediated reactions. The genes coding for the primary enzymes involved in the biosynthesis of PHA are the *phaA* (β-ketothiolase), *phaB* (acetoacetyl-CoA reductase), and *phaC* (PHA synthase) genes [50], which commonly serve as a screen for microbial PHB production capacity as they are relatively well conserved [51,52]. Typically, proteins known as phasins accumulate during PHA synthesis, which bind PHA granules and promote further PHA production [53]. PHA synthases use coenzyme A (CoA) thioesters of hydroxyalkanoic acids (HAs) as substrates and catalyse the polymerisation of HAs into PHA with the concomitant release of CoA. Although PHB can function as a sole growth substrate in aerobic cultures enriched on acetate during periods of carbon deficiency, in an elegant set of experiments using a methanotrophs (*Methylocystis parvus* OBBP), PHB was used as a source of reducing power to aid methane consumption, as opposed to the supply of C_2_ units for synthesis [54].

PHB synthesis in methylotrophs, such as *Methylobacter extorquens* [55,56], and methanotrophs [35,36,37,38] is well documented for C_1_ substrates, such as methane or methanol. In the seminal paper on PHB production by methanotrophs, Asenjo and Suk [38] analysed the biochemical pathways in type II methanotrophs to establish the preliminary kinetic analysis and stoichiometry of PHB synthesis. Their overall equation for PHB accumulation for methanotrophs using the serine pathway was presented as:

8 CH_4_ + 12 O_2_ + FP → C_4_H_6_O_2_ (PHB monomer) + 4 CO_2_ + 12 ATP + FPH_2_

where FP = oxidised succinate dehydrogenase, and FPH_2_ = reduced succinate dehydrogenase.

Although the theoretical mass yield for this bioconversion based on this equation is 67% (86 g PHB/128 g methane), this excludes the fraction of the methane and oxygen that has to be converted to CO_2_ to regenerate the NADH required for biosynthesis. Wendlandt *et al.* [27,37,57] obtained yields of 0.55 g PHB/g methane in an enriched (>90%) culture of *Methylocystis* sp. GB 25 DSMZ 7674, close to the theoretical maximum, with PHB contents in the biomass of 51 wt%. In this series of studies, the methane-utilizing mixed culture was maintained under non-sterile found to have the potential of self-regulation resulting in a stable composition even under such non-aseptic conditions [58]. However, Zúňiga *et al.* [59] reported higher yields of 0.81 g PHB/g methane (reported as 0.60 g carbon from PHB with respect to g carbon in methane consumed) for a mixed methanotrophic community including a *Methylobacterium organophilum* strain, although with only 34% PHB content overall. The isolated strain by itself, though, had up to 58% PHB content after accumulation at a mass yield of 0.59 g PHB/g methane. 

Most quantitative PHB production studies are reported from Type II *α*-proteobacter genera, such as *Methylocystis* and *Methylosinus*, *Methylococcus*, and *Methylomonas* spp. [38], with molecular weights (*M_w_*) up to 3.1 MDa being recorded [37]. For example, Asenjo and Suk [38] reported PHB production of up to 67% of cell dry weight using a pure culture of *Methylosinus trichosporium* OB3b—the highest PHB content reported to date from methane. Overall, total PHB contents obtained for accumulations from pure and mixed methanotrophic cultures are summarised in Table 1; less data is available for mass or carbon yields in these systems. While these concentrations of PHB are lower than those produced in bacteria that feed on sugars, they are still significant and show promise for industrial applications. 

Although qualitative data regarding PHB production has been reported in RuMP-pathway γ-proteobacteria [60,61,62], production is still uncertain [63]. Previous methylotroph studies suggest that PHB synthesis may be linked to the serine cycle, and that RuMP-pathway methanotrophs may be incapable of PHB synthesis. An early study detected no 3-ketothiolase, acetoacetyl-coenzyme A reductase or PHA synthase in RuMP-pathway methylotrophs, and none of the bacteria produced a measurable amount of PHB [64]. Pieja *et al.* [63] evaluated twelve strains from six different genera of methanotrophs for encoding of PHB synthase (*phaC*), as well as PHB production under nitrogen-limited conditions in γ-proteobacter and α-proteobacter methanotrophs. The γ-proteobacter strains tested negative for *phaC* gene, as well as PHB production, while all α-proteobacter strains tested positive for *phaC* and PHB synthesis. Babel [65] hypothesised that serine-pathway methylotrophs produce PHB as a carbon storage polymer, whereas RuMP-pathway methylotrophs produce exopolysaccharides under unbalanced growth conditions. More recent research suggests that PHB accumulation is associated with the supply of reducing equivalents, rather than providing cell components during growth [54].

**Table 1:** Summary of the methylotrophic and methanotrophic production of PHB. Adapted from Khosravi-Darani *et al.* [66] and Karthikeyan *et al.* [6].

Microorganisms (% in Mixed Culture)	Carbon Source	PHB Content (% of Total Biomass)	Yield (g PHB/g Carbon Source)	References
*Methlocystis* sp. GB25 DSMZ 7674 (>90%)	Methane	28.3–51.3	0.55	[27,57,58]
*Methlocystis* sp. GB25 DSMZ 7674 (>86%)	Methane	10.4–33.6	0.45	[37]
*Methylosinus/methylocystis* (percent unknown) + Type I genera	Methane	7–46	n/a	[63]
Mixed consortium including *Methylobacterium organophilum* (percent unknown)	Methane	34	0.80*	[59]
*Methylosinus* and *Methylocystis* spp. dominant in mixed consortium (percent unknown)	Methane	17–26	n/a	[67]
*Methylocystis* (~77%)	Methane	39	0.64 *	[68]
*Methylocystis* (> 76%)	Methane	40	0.49 *	[69]
*Methylomicrobium* (96%)	Methane	23	n/a	[70]
*Methylocystis* sp. (71.6%)	Methane	2.5–8.5	n/a	[71]
*Methylosinus trichosporium*	Methane	20–25	n/a	[72]
*Methylocystis parvus* OBBP	Methane	68	n/a	[38]
*Methylosinus trichosporium* OB3b	Methane	30–50	n/a	[73]
Type II methanotrophic strain MTS	Methane	3	n/a	[35]
*Methylosinus trichosporium* OB3b	Methane	30	n/a	[74]
**Microorganisms (Pure Culture)**	**Carbon Source**	**PHB Content (% of Total Biomass)**	**Yield (g PHB/g Carbon Source)**	**References**
*Methylosinus trichosporium* OB3b *Methylobacterium organophilum* strains	Methane Methane	25 38–57	n/a 0.48–0.59 *	[59]
*Methylocystis* spp. *Methylosinus* spp.	Methane Methane	7–36 9–38	n/a n/a	[63]
*Methylocystis hirsute*	Methane	51.6	n/a	[75]
*Methylocystis parvus* OBBP *Methylosinus trichosporium* OB3b	Methane	60 29	0.88 1.13	[76]
*Methylocystis parvus* OBBP	Methane	49.4	n/a	[77]
*Methylosinus trichosporium* IMV3011	Methane + methanol	40	n/a	[36]
*Methylosinus trichosporium* IMV 3011	Methane + Methanol	46	n/a	[78]
*Pseudomonas* sp. K	Methanol	66	0.18	[79]
*Methylobacterium rhodesianum*	Methanol	45–55	n/a	[65]
*Methylobacterium extorquens* K *Paracoccus denitrificans*	Methanol + *n*-amyl alcohol	44 57	0.11 (0.97 on *n*-amyl alcohol)	[80]
*Pseudomonas 135*	Methanol	55	n/a	[81]
*Methylobacterium extorquens NCIMB 9133*	Methanol	7–21		[82]
*Methylobacterium extorquens ATCC 55366*	Methanol	40–46	0.09–0.12	[55]
*Methylobacterium organophilum*	Methanol	52–56	0.19	[83]
*Methylobacterium sp V49*	Methanol	11	n/a	[84]
*Methylobacterium extorquens* AM1	Methanol	34–42	n/a	[85]
*Methylobacterium extorquens* AM1	Methanol	22–25	n/a	[56]
*Methylobacterium sp.* GW2	Methanol	40	n/a	[86]
*Methylobacterium extorquens* DSMZ 1340	Methanol	35	0.3	[87]
*Methylosinus trichosporium* IMV 3011	Methanol	32	n/a	[88]
*Methylobacterium extorquens* AM1	Methanol	27	n/a	[89]
*Methylobacteria extorquens* G10 *Methyloligella halotolerans* C2	Methanol	40 17	n/a	[90]

* Based on reported yields of g carbon from PHB with respect to g carbon from methane or mole carbon from PHB with respect to mole carbon from methane

## 3. Process Conditions for PHB Production from Methane

The effects of operating conditions on PHB production using methanotrophs have been reviewed in some detail [6,66]. In summary, PHB production is typically achieved using a two stage process, with an initial period of cell growth under nutrient-sufficient conditions, then PHB accumulation is triggered by the absence of one or more major nutrients needed for cell division (unbalanced growth), with this being exaggerated under conditions of transient nitrogen and methane availability [69,91]. In mixed cultures, this two-stage process can be repeated multiple times to select for PHA accumulators. 

There are a great many studies that have explored the relative merits of mixed cultures *versus* pure strains for PHA accumulation in general, including some that are specific to the use of methane as a carbon source [14,58,70,91,92,93,94]. Overall, these studies suggest that such mixed cultures may provide specific benefits such as: co-culture bacteria removing potentially toxic byproducts (e.g., methanol or formaldehyde) from the medium; beneficial nutrients being supplied by co-culture bacteria; operation under non-sterile conditions; and even possibly higher yields and production rates. However, many questions still remain with respect to optimizing the conditions for PHA production and culture selection. 

It is known, for example, that the use of relatively high dissolved oxygen (DO, 9 mg/L) and nitrate as the nitrogen source will favour the growth of the Type I γ-proteobacteria [67], while the use of dissolved nitrogen as the nitrogen source, with a low influent DO (2 mg/L) was found to shift the culture to Type II α-proteobacteria [67], although the *Methylocystis* species may be an exception to this, tolerating a wide range of CH4/O2 ratios [95]. It is also known that nutrient concentration affects γ-proteobacteria and that they prefer a neutral pH, while α-proteobacteria are typically more acidophilic and, thus, acid-tolerant [63,67,96,97,98]. In pure strains, methanotrophic acitivity can be inhibited at DO levels >1 mg/L [99]. The addition of carbon dioxide as a gas or as bicarbonate may increase productivity [100], while iron deficiency can have a negative impact [37]. Metal ions such as copper, nickel, and zinc are important in the regulation of the activity of the pMMO and sMMO enzymes [101,102], with inhibitory effects on sMMO under some concentrations, while potassium deficiency has been shown to cause increased molecular weight in a *Methylocystis* dominated mixed culture [37]. Overall, there are many critical factors that affect PHA accumulation in methanotrophic bacteria, and these need to be carefully controlled. 

According to the literature to date, the gases that make up biogas and natural gas do not inhibit the bacteria [75,92]. Natural gas is a mixture of several hydrocarbon gases containing predominantly methane (80%–95%), and may contain other heavier alkanes such as ethane, propane, butane and pentane [39]. Typically, biogas contains primarily methane (60%–70%) and carbon dioxide (30%–40%), with traces of nitrogen and hydrogen sulfide [103]. The CO_2_ in the biogas may even assist the growth of α-proteobacterial (PHB producing) methanotrophs, as described above, as they use CO_2_ in the serine cycle. For the most part the α-proteobacteria methanotrophs are also more resilient to gas fluctuation and variation than the γ-proteobacterial methanotrophs, since they are most robust to higher methane concentrations [104]. 

There have been a wide range of reactor types used in the laboratory scale production of PHB from methane. In the majority of cases, simple batch reactors (such as bottles, shake flasks, tanks, or fermenters) have been used, with either a static head or flow through of gas and under static, shaken or stirred conditions. On a micro scale, Sundstrom and Criddle used a high-throughput microbioreactor with aerated microtiter plates for rapid screening and isolation [77]. On a larger lab scale, pressurised bioreactors were used by Wendlandt *et al.* [27] while Zúñiga *et al.* [105] used a two-phase partitioning bioreactor, Rahnama *et al.* explored the use of a bubble column and vertical loop reactor [75], and Pfluger *et al.* adapted a laboratory scale fluidized bed reactor for growth of methanotrophs [67]. All of these processes were found to be viable, although none at this stage appear to show very significant improvements in productivity relative to the others. 

The scale-up of biological processes is never a simple process. In the case of gas-liquid transfer reactions, there are a variety of reactor types available, ranging from continuous stirred tank reactors (CSTRs) to bubble lift and airlift reactors. CSTRs do not scale up well for gas fermentation, and airlift reactors are generally employed at scale, frequently using static mixers to ensure gas distribution [106,107]. Companies such as Calysta (Menlo Park, CA, USA) that use methane, or Lanzatech (Skokie, IL, USA) that use syngas, have spent considerable effort enhancing fluid dynamics and mass transfer of gases into the liquid phase. Detailed process and plant options from Unibio A/S website indicate the use of a U-tube reactor with a significant headspace (essentially an airlift reactor where the downcomer is equal in proportion to the riser) that has been implemented in a pilot-scale plant in Trinidad and Tobago—which uses natural gas as a methane source for single-cell protein production. The economic feasibility of PHB production from methane was first reported by Listewnik *et al.* [92]. They studied a relatively small-scale process (500 t/a) and found that the cost of production of PHB from methane at this scale was of the order of $8.5/kg. Since that time, Newlight Technologies have also commercialised a proprietary greenhouse gas-to-plastic technology, ramping up from pilot to commercial scale production in 2013. Newlight Technologies has added 22.7 thousand t/a production capacity (and signed an 8.6 million tonne offtake agreement with Vinmar), although process details for this technology are unavailable [108]. Likewise Mango Materials uses proprietary technologies to produce PHB from waste biogas (methane) in a process that is claimed to be economically competitive with conventional oil-based plastics [109].

In a recent paper, a techno-economic assessment of the potential for the production of 100,000 t/a of PHB from methane with at least 98% purity was undertaken [28]. Capital and operating costs were estimated, with air-lift bioreactors with a concentric internal draft tube being selected to reduce mixing costs at this large scale. The surface area to volume ratio is restricted at this scale, limiting the potential for heat removal so that heat removal from a two-stage bioreactor process running at 38 °C contributed 28% of the overall operating cost. Energy consumption for air compression and biomass drying were also identified as significant capital and operating costs, and the effects of bioreactor height and pressure and biomass moisture content need to be understood. 

In designing such processes, one also needs to consider that while the use of air is frequently employed at lab scale, at an industrial scale this is problematic because 78% of the gas volume is wasted (unnecessary gas holdup volume) and the effectively dilute oxygen (21%) is much less efficient for mass transfer. Pure oxygen is expensive to provide at an industrial scale. Oxygen-enriched air can provide a higher concentration gradient between the gas and liquid phase (and occupy less gas volume in the reactor), but has greater capital costs than an air compressor. Thus, it is also necessary to consider the cost of supplying oxygen (up to half the total production cost), the hazardous nature of combining explosive/flammability gases, the risk of culture contamination affecting the culture stability and biopolymer yield/characteristics, as well as culture instability at low oxygen supply [24]. The downstream isolation technologies also need to be optimised, with many options having been proposed in the literature, including bead milling, high-pressure homogenisation, flotation, supercritical fluid extraction, chemical and enzymatic digestion, and solvent extraction [110].

## 4. High-Performance Biomaterials from Methane: PHA Co-Polymers

As discussed, many methanotrophic bacteria are known to synthesise PHB under N-limited conditions, with methane as the sole carbon source. PHB is an attractive polymer for many reasons, including its inherent biodegradability in aerobic and anaerobic environments, particularly marine, its water resistance, biocompatibility, optical purity, and piezoelectric properties [1]. However, the range of applications for PHB is currently limited due to its stiffness and brittleness, caused by its high crystallinity and also large spherulite size (if not processed to minimise this). This is reflected particularly in its elongation to break, which is around 100 times less than that of low density polyethylene [1]. In addition, the high melting temperature for PHB is matched by a low degradation temperature for the untreated polymer without stabiliser. This means that it is difficult to process while retaining material properties. Despite these limitations, a number of companies have commercialised the production of PHB, with applications in many areas including: biomedical devices, controlled-release and drug delivery applications, and injection molded articles, such as bottles, food containers, and cutlery, and many others [111]. 

Co-polymerisation of 3-hydroxybutyrate (3HB) with alternative hydroxyalkanoate (HA) monomers during biological synthesis is one alternative to generate biopolymers with more desirable properties. Through this approach, PHAs with different and tailored mechanical properties can be made, ranging from hard and crystalline to tough and flexible to elastic and rubbery. Again this is an approach that has been adopted by commercial manufacturers, using pure strains and sugar-based feedstocks. An example of such a copolymer is poly(3-hydroxybutyrate-*co*-3-hydroxyvalerate) (PHBV), a polymer potentially tougher and an order of magnitude more elastic than PHB (Table 2). The lower melting temperature of PHBV, which is matched with the same degradation temperature as for PHB, also means that these polymers are much more readily processed without loss of properties. 

**Table 2:** Properties of PHB compared to PHBV and other PHAs [112]

Polymer	Melting Temperature *T_m_* (◦C)	Glass-Transition Temperature *T_g_* (◦C)	Young’s Modulus (GPa)	Tensile Strength (MPa)	Elongation to Break (%)
PHB	180	4	3.5	40	5
P(3HB-*co*-20 mol%3HV)	145	−1	0.8	20	50
P(3HB-*co*-6 mol%3HA) *	133	−8	0.2	17	680
Polypropylene	176	−10	1.7	38	400
Low-density polyethylene	130	−30	0.2	10	620

* HA, mixed hydroxyalkanoate co-monomer units including 3-hydroxydecanoate (3 mol%), 3-hydroxydodecanoate (3 mol%), 3-hydroxyoctanoate (1 mol%), 3-hydroxy-cis-5-dodecenoate (1 mol%).

## 5. Strategies for Producing PHA Co-Polymers from Methane

There are various potential strategies for directly converting methane into more versatile PHAs (such as PHBV copolymers) using biological means. The growth media can be supplemented with a co-substrate that is directly metabolised to form a fatty acid-CoA, such as propionyl-CoA, which is then condensed with acetyl-CoA to form starting materials for copolymer synthesis. In the case of propionyl-CoA, 3-ketovaleryl-CoA is formed which, in turn, is condensed with 3-hydroxybuturyl-CoA to form the biopolymer PHBV. Another potential route involves a co-culture/mixed culture where the non-methanotrophic strain is capable of generating a PHA, while surviving off a carbon source that is essentially a byproduct of methanotroph metabolism. Alternatively, there are various combinations of biological and chemical means that could be integrated to yield more attractive PHA co-polymers and their derivatives. These strategies are not without their challenges and are briefly summarised in Table 3 and discussed further in this section.

### 5.1. Direct Methanotrophic PHA Synthesis by Adding External Precursors

A copolymerisation strategy relying on the addition of precursor compounds that are structurally related to the desired co-monomer units is possible and well-established. Ueda *et al.* [80] supplemented a methanol feedstock with a 3HV precursor, n-amyl alcohol (a C_5_ alcohol), to synthesise a PHBV copolymer using methylotrophs (*Paracoccus denitrificans* and *Methylobacterium extorquens*). Babel used fructose as the co-substrate in another early study using methanol for PHA production from *Methylobacterium rhodesianum* and *M. extorquens* producing, in some cases, very high proportions of HV in the copolymers produced [65]. Yezza *et al.* [86] likewise produced PHBV at 67 mol% HV content using valeric acid as a co-substrate with methanol in *Methylobacterium* sp. GW2. Orita *et al.* [89] were able to use cobalt deficient conditions to synthesis PHBV from methanol alone. 

The successful production of PHBV copolymers from methane was also demonstrated by Zúñiga *et al.* [105] in *Methylobacterium organophilum* CZ-2 using citrate or propionate as a co-substrate. The largest PHA yield to date (at 82% w/w) was obtained with citrate as a co-substrate, and had a 3HB:3HV:3HO ratio of 86:14:0, where 3HO is 3-hydroxyoctanoate. An 80% *w/w* yield was also obtained using propionate as a co-substrate, with the copolymer having a 3HB:3HV:3HO ratio of 70:30:0. NMR analyses revealed six different monomers with citrate in the media (3HB, 3HV, 4-hydroxyvalerate, 4-hydroxyhexanoate, 3HO, and 4-hydroxyoctanoate), indicating a remarkable diversity in the PHA produced by this methanotrophic strain. Myung *et al.* [69] recently demonstrated the potential to tailor poly(3-hydroxybutyrate-*co*-3-hydroxyvalerate) from methane using an enriched culture dominated by a *Methylocystis* species under non-aseptic conditions. When fed CH_4_ plus valerate, PHBV was synthesized. As expected, the mol% of 3HV increased with additional valerate. Adding different HAs also allowed the tailoring of PHA copolymer composition in an enriched culture of methanotrophs and in two pure strains (*Methylocystis parvus* OBBP and *Methylosinus trichosporium* OB3b) [113]. Only PHB was synthesised when using methane as the carbon source. Adding 3-hydroxybutyric acid to the media increased the PHB yield; adding propionate led to PHBV synthesis; and including valerate further increased PHBV content, with higher levels of valerate being associated with higher 3HV contents in the copolymer produced [113]. While this strategy has proven successful and would seem compatible with generating PHBV using a mixed consortium, the addition of these precursors is an additional cost.

The use of alternating carbon feed sources to produce block copolymers has also been demonstrated in pure and mixed cultures [114] and represents a route to novel materials with unique microstructure and material properties. This could be an interesting process to assess, using methane as the primary substrate, alternating it with other C_1_ compounds such as methanol or formate, or C_3_ compounds, such as propionate or propanol. Again, the addition of other carbon sources represents an additional cost that would have to be balanced against the value of the copolymer production yields and rates.

### 5.2. Indirect Biological PHA Production: Synthesis in Consortia

Aerobic methanotrophs naturally exist in microbial consortia, where carbon derived from methane is distributed to non-methanotrophic heterotrophs [32]. In natural ecosystems and laboratory enrichment cultures, methane assimilation and distribution has been observed to support a diversity of microbes that may comprise a complex, multi-tiered microbial food web [115]. Kalyuzhnaya *et al.* [46] demonstrated that methane assimilation using a highly efficient pyrophosphate-mediated glycolytic pathway under low oxygen tension. Here, a methanotroph (*Methylomicrobium alcaliphilum* 20Z) secreted fermentation products such as lactate, acetate, and H_2_ under low oxygen tension, all potential metabolites that could be used by other bacteria within the consortia. A striking example of this symbiotic mechanism occurs within the consortia in reactors used to produce single cell protein. The stability of continuous cultures of a methanotroph (*Methylococcus capsulatus* str. Bath) is dependent on the consortium to maintain stability [116].

Various bioreactors of single-cell protein-producing *Methylococcus capsulatus* Bath were consistently invaded by three bacteria: an *Aneurinibacillus* species, a *Brevibacillus* agri strain, and a *Ralstonia* species [116]. The *Ralstonia* species is of particular significance as this acetate-oxidising bacterium is a known PHA accumulator. *Ralstonia eutropha* produces both the homopolymer PHB [117]. When provided with the appropriate substrate, it can produce PHBV. When levulinic acid was added to a *Ralstonia eutropha* culture, the 3HV content in PHBV was up to 41% [118]. 

A low-substrate-specificity PHA synthase *Pha*C2_Ps_ was expressed in a PHB negative *Ralstonia eutropha* strain, allowing it to accumulate short and medium chain length PHA copolymers when grown on mixed carbon sources [119]. This suggests that mixed culture used for single cell production may also serve as a feasible example for co-cultures that generate PHBV from methane-derived carbon. The methanotrophs would provide the carbon for *Ralstonia*, and imbalanced growth with HA supplementation could facilitate copolymer production. Although feasible, the yields may be low as the PHBV content would be a fraction of *Ralstonia* sp. and other biomass in the mixed population.

### 5.3. Downstream Processing of PHA—Including Precursor Production from Methane

There is also significant potential for value-adding by broadening the range of products derived from PHAs through further downstream product development. Polyhydroxyalkanoates are readily modified, and processes, such as grafting other functional groups onto the polymer chain, are well-established [120,121,122]. Blends, composites and highly-elongated fibres of unique strength and toughness have all been well studied [123,124]. The production of oligomeric blocks of PHA also provides a valuable route to alternative materials, such as synthetic block copolymers and other novel structures [125,126].

An alternative strategy for biopolymer synthesis is to produce other types of biologically-derived monomers or oligomers from methane, which may be subsequently polymerised or synthetically-coupled to PHA or its derivatives into a material of choice. A current example is the production of lactic acid as a precursor for chemical synthesis. Calysta have genetically modified a methanotroph with an exogenous nucleic acid that expresses lactate dehydrogenase. This delivers significantly enhanced lactate production from the reverse reaction, where pyruvate is converted to lactate [127], while presumably regenerating reducing equivalents (NAD^+^ is reduced to NADH) in the catalysis. This process is currently being commercialised by Calysta in conjunction with Natureworks in the US. 

### 5.4. Synopsis

The options discussed in this article are summarised in Table 3. Each process has inherent restrictions, some of which are unknown and will require further research to elucidate. While the production of PHBV using methanotrophs is established, the decreased substrate cost still needs to be balanced against provision of other carbon sources/supplements, yield, production rate, extraction and processing, and capital expenditure for bioreactors and gas provision. Establishing the range of these values will provide clarity as to where improvements are required to progress towards financial viability for this versatile biopolymer.

**Table 3:** Strategies for PHA and PHA derivative production using methane as the primary substrate.

Process	Pros	Cons	Unknowns
Direct production of PHB from CH_4_	Has been achieved	Average to poor mechanical properties	Processes for maximising yields and rates
Methanotroph PHBV production through the co-addition of a fatty acid with odd carbon numbers	Has been achieved	The cost of the C_odd_ feedstock offsets the savings of CH_4_; may produce blends of polymer product if there is variation in uptake rates amongst the different members of the community	Yields, homogeneity and maximum co-monomer content
Methanotrophs facilitating growth of a co-culture capable of PHBV production (direct or co-substrate addition)	Potentially achievable using *Ralstonia* sp.	The cost of the C_odd_ feedstock offsets the savings of CH_4_; may produce blends of polymer product if there is variation in uptake rates amongst the different members of the community	Yields, homogeneity and maximum co-monomer content
Generate alternative copolymers through supply of appropriate feed.	Delivers broader range of mechanical properties	Cost of adding the monomers	Yields, homogeneity and maximum co-monomer content
Use a co-feeding strategy of timed pulses of methane and alternative feeds to tailor copolymer compositional distribution	Tailored, e.g., block copolymers already produced in the literature using alternating feeding strategy	Not proven in methanotrophs. May produce blends	Pulses of gas feed alternating with soluble carbon feed may prove difficult for cells to adapt to
Generate monomers biologically and polymerise *ex situ*	Can achieve desired copolymer composition	Monomer concentration and purification	Currently being commercialised
Downstream polymer modification/functionalisation/ depolymerisation into oligomers and use as building block	Processes are well established	Costly, intensive additional processes and can be time-consuming	Potential for development of unique material properties for niche applications.

## 6. PHA Production from Other Gas Streams

It should be noted that methane is not the only gaseous feedstock that can be considered for PHA production using microbes. As mentioned in the introduction, syngas (a gas mixture consisting primarily of hydrogen, carbon monoxide, with potentially some carbon dioxide) is well known as a feedstock for PHA production, with a techno-economic analysis having been recently conducted on a gasification-based hybrid biorefinery producing both hydrogen gas and PHA [128]. Hydrogen-oxidising bacteria (or Knallgas bacteria) have the ability to utilize hydrogen as an electron donor and oxygen as an electron acceptor to fix CO_2_ via the ribulose biphosphate or reverse tricarboxylic cycle [129]. *Cupriavidus necator* is one example of such an organism that is well known for producing PHA [130]. In addition, many cyanobacteria have the capacity to produce PHA from CO_2_, in the presence of light, although additional nutrients accelerate the production rates and at this stage the process is not economic [131].

## 7. Conclusions

Over the past decades, the production of PHA from methane by methanotrophic bacteria and the study of their PHA biosynthesis pathways were generally limited to pure cultures. The most widely produced PHA is the homopolymer PHB, but this is a low performance biopolymer that is highly crystalline and brittle, with poor elastic properties that limit the process and end-use applications. The copolymer PHBV can be tougher and more elastic with the correct composition and microstructure. Potentially, PHBV copolymers could be produced from mixed cultures of methanotrophic bacteria using methane as a feedstock, reducing the production costs associated with substrate and reactor sterility. However, the production of a consistent PHBV copolymer from methane is not a straightforward process. We present production scenarios that include (1) enriching a consortia, or using a stable mixed culture, and supplementing the feed with C_3_ or C_5_ co-substrates that could be used directly by the methanotrophs or (2) enriching their consortia with known PHBV accumulators such as *Ralstonia* spp. and (3) alternating the feed regime between methane and an alternative C source to tailor the desired copolymer, or (4) use methane to generate copolymer precursors that are synthetically assembled into the desired co-polymer. A fundamental understanding of polymer properties from methanotrophic mixed cultures is necessary, as it is important to characterise the relative purity of the product within the polymer blend, as this significantly affects polymer properties. Additional research is still required to establish the feasibility and shortcomings of ideas presented in this article.
